# DNA methylation in ELOVL2 and C1orf132 correctly predicted chronological age of individuals from three disease groups

**DOI:** 10.1007/s00414-017-1636-0

**Published:** 2017-07-19

**Authors:** M. Spólnicka, E. Pośpiech, B. Pepłońska, R. Zbieć-Piekarska, Ż. Makowska, A. Pięta, J. Karłowska-Pik, B. Ziemkiewicz, M. Wężyk, P. Gasperowicz, T. Bednarczuk, M. Barcikowska, C. Żekanowski, R. Płoski, Wojciech Branicki

**Affiliations:** 1 Central Forensic Laboratory of the Police, Aleje Ujazdowskie 7, 00-583 Warsaw, Poland; 2 Department of Genetics and Evolution, Institute of Zoology of the Jagiellonian University, Gronostajowa 9, 30-387 Krakow, Poland; 3 Malopolska Centre of Biotechnology of the Jagiellonian University, Gronostajowa Gronostajowa st. 7A, 30-387 Krakow, Poland; 4 Laboratory of Neurogenetics, Department of Neurodegenerative Disorders, Mossakowski Medical Research Center, Polish Academy of Sciences, 02-106 Warszawa, 5 Pawinskiego St, Warsaw, Poland; 5 Faculty of Mathematics and Computer Science, Nicolaus Copernicus University, Chopina 12/18, 87-100 Toruń, Poland; 6 Department of Medical Genetics, Centre for Biostructure, Medical University of Warsaw, Pawińskiego 3c, 02-106 Warsaw, Poland; 7 Department of Internal Medicine and Endocrinology, Medical University of Warsaw, Banacha 1a, 02-097 Warsaw, Poland

**Keywords:** DNA methylation, Chronological age, Alzheimer’s disease, Graves’ disease, Neural networks, Prediction accuracy

## Abstract

Improving accuracy of the available predictive DNA methods is important for their wider use in routine forensic work. Information on age in the process of identification of an unknown individual may provide important hints that can speed up the process of investigation. DNA methylation markers have been demonstrated to provide accurate age estimation in forensics, but there is growing evidence that DNA methylation can be modified by various factors including diseases. We analyzed DNA methylation profile in five markers from five different genes (ELOVL2, C1orf132, KLF14, FHL2, and TRIM59) used for forensic age prediction in three groups of individuals with diagnosed medical conditions. The obtained results showed that the selected age-related CpG sites have unchanged age prediction capacity in the group of late onset Alzheimer’s disease patients. Aberrant hypermethylation and decreased prediction accuracy were found for TRIM59 and KLF14 markers in the group of early onset Alzheimer’s disease suggesting accelerated aging of patients. In the Graves’ disease patients, altered DNA methylation profile and modified age prediction accuracy were noted for TRIM59 and FHL2 with aberrant hypermethylation observed for the former and aberrant hypomethylation for the latter. Our work emphasizes high utility of the ELOVL2 and C1orf132 markers for prediction of chronological age in forensics by showing unchanged prediction accuracy in individuals affected by three diseases. The study also demonstrates that artificial neural networks could be a convenient alternative for the forensic predictive DNA analyses.

## Introduction

Forensic intelligence through DNA analysis is now achievable when searching for an unknown individual in criminal, identification, or security cases. Wider use of the predictive DNA analysis methods in forensic investigations depends largely on their accuracy. Currently available predictive tests include biogeographic ancestry, pigmentation, hair loss and hair shape, extreme stature, facial morphology, and age [1]. All these methods are still being revised by using more advanced mathematical approaches, detailed studies of molecular mechanisms involved in phenotype determination and selection of additional predictors [2–6].

Age prediction has an important place in predictive DNA analysis. Informative itself, it can also increase prediction accuracy of several progressive physical appearance traits. Particularly, prediction of such appearance traits like hair graying, baldness, or skin wrinkles can be feasible only if biological age is included in prediction modeling.

Recent progress in epigenomics allowed identification of multiple DNA methylation loci that can be useful for age prediction [7–9]. Some of these markers have been used to develop age prediction models that may have a practical value in forensics [7, 10–13]. It has been shown that DNA methylation markers outperform the other types of potential age predictors including age-shortening telomeres, age-dependent changes in T cells’ DNA, and age-altering mRNA level [14]. However, their use in routine forensic work still needs to be confirmed since prediction outcome can potentially be influenced by aberrations of DNA methylation patterns caused by various factors. It is worth noting that many of the identified DNA methylation markers used in age prediction are located in genes with known functions in Alzheimer’s disease, cancer, tissue degradation, DNA damage, and oxidative stress [8]. There is growing evidence that factors like tobacco, alcohol, carcinogens, stress or infectious diseases, and even diet or physical activity can influence DNA methylation [8, 15]. The potential age predictors may show different sensitivity to the influence of environment, medical history, or life style and thus show various capabilities to predict chronological age. Notably, Marioni et al. showed that faster aging predicted from DNA methylation status is heritable and predicts mortality independently of environmental or genetic factors [16]. Further research should explain whether particular DNA methylation markers might get misaligned as age predictors in individuals suffering from particular diseases or other external factors and thus be less suitable for accurate prediction of chronological age in diseased individuals. Indeed, it has been shown that DNA methylation status in some loci may depend on the number of cell divisions or can be influenced by other factors [17]. Thus, studies aiming to identify all potential players influencing differences in DNA methylation at particular loci between individuals at the same chronological age are important for better understanding the correlation between DNA methylation and age as well as for better accuracy of age prediction models. Exploration of this issue is important for age prediction reliability in routine forensic investigation.

In this study, we address the problem of age prediction accuracy of DNA methylation markers. We test DNA methylation status and prediction capacity of five literature age prediction markers studying three groups of individuals with different conditions including patients of early onset Alzheimer’s disease (EOAD), late onset Alzheimer’s disease (LOAD), and Graves’ disease (GD). Our main motivation to study these groups was that all three diseases might potentially be associated with accelerated aging and affect age prediction accuracy parameters and thus perfectly fit with our aim to evaluate potential dysregulation of DNA methylation of markers used for chronological age prediction.

## Material and methods

### Study samples and the protocol

Written informed consent was obtained from AD and GD patients (or their legal representatives) and controls, according to the Declaration of Helsinki (BMJ 1991; 302:1194). The genetic study was approved by the Ethics Committee of the CSK-MSW Hospital (Warsaw, Poland) in compliance with national legislation and the Code of Ethical Principles for Medical Research Involving Human Subjects of the World Medical Association and at the Institute of Cardiology in Warsaw, no. IK-NP-0021-79/1396/13. Peripheral blood collected in EDTA-containing tubes was analyzed from 31 EOAD patients, 68 LOAD patients, and 91 GD patients. Detailed information about the tested groups is given in Table 1. Total DNA was extracted from the obtained blood samples using standard salting out procedure [18], the phenol/chloroform method [19], or PrepFiler kit according to the manufacturer’s directions (Applied Biosystems, Foster City, CA). The total number of five CpG sites in five genes (ELOVL2, C1orf132, KLF14, TRIM59, and FHL2) were analyzed in the three groups using pyrosequencing technology. One to two micrograms of DNA was subjected to bisulfite conversion using the EpiTect 96 Bisulfite Kit according to the manufacturer’s protocol (Qiagen, Hilden, Germany), and then, the previously applied PCR and sequencing protocols were used to measure DNA methylation status of the studied cytosines. In addition, DNAm data for 425 samples examined in our previous study [11] were used as a training set (305 samples) and healthy controls’ testing set (120 samples).

**Table 1:** Characteristics of the testing set groups

Tested groups	Number	Mean Age ± SD	Min age	Max age	Male (%)
Healthy controls [11]	120	41.1 ± 20.2	2	75	47.5
Late onset Alzheimer’s disease (LOAD)	68	70.9 ± 3.4	65	75	44.1
Early onset Alzheimer’s disease (EOAD)	31	44.2 ± 10.2	31	68	48.4
Graves’ disease (GD)	91	44.4 ± 22.1	12	76	34.1

### Statistics

#### DNA methylation profile in individuals from the tested disease groups

DNA methylation percentage measured for five age-related CpG sites (ELOVL2 c7, C1orf132 c1, FHL2 c2, TRIM59 c7, KLF14 c1) was compared between individuals from three disease groups and matched healthy controls using independent sample Student’s *t* test. Because of the known differences in DNAm age correlation and age prediction accuracy between younger and older individuals [e.g., 11, 13], patients were divided into age group categories and calculations were performed for each age group separately. EOAD patients were divided into younger EOAD group (age 31–44) and older EOAD group (age 45–68); GD patients were categorized into younger GD group (age 12–30) and older GD group (56–76), while all LOAD patients accounted for only one age group category (age 65–75). Age categories are different for particular diseases due to different numbers of individuals at particular ages in various disease groups. Sizes of particular age group categories are provided in Table 2. The healthy controls were selected from the available set of 120 controls taken from our previous study [11] and matched separately for the particular groups of EOAD, LOAD, and GD patients using criteria of mean age and age distribution between the compared groups. A proper distribution of age in the disease groups and healthy controls was confirmed with nonparametric Kolmogorov-Smirnov test. Analyses were performed using PS IMAGO 4 (IBM SPSS Statistics 24).

**Table 2:** DNA methylation status and age prediction accuracy of single age-related CpG sites measured in GD, EOAD, and GD patients compared to age-matched healthy controls

Locus CpG site	Disease group	Age group	No. of patients	No. of controls	Mean % of DNA methylation			MAE of predicted and chronological age		
					Patients	Controls	*P* value	Patients	Controls	*P* value
ELOVL2 c7	GD	Younger	44	39	60.34	57.59	0.055	3.918	3.861	0.935
		Older	47	33	83.43	82.76	0.529	6.390	7.960	0.299
		Total	91	72	72.26	69.13	0.136	5.195	5.740	0.522
	EOAD	Younger	17	27	74.18	70.96	0.137	7.704	7.330	0.877
		Older	14	30	74.36	79.33	0.253	7.285	4.522	0.100
		Total	31	57	74.26	75.37	0.566	7.530	5.852	0.237
	LOAD	Total	68	28	83.32	84.00	0.465	11.912	9.114	0.075
C1orf132 c1	GD	Younger	44	39	81.89	81.33	0.633	3.704	4.935	0.159
		Older	47	33	54.46	51.53	0.083	10.765	9.220	0.380
		Total	91	72	68.20	67.33	0.732	7.351	6.899	0.672
	EOAD	Younger	17	27	68.94	68.26	0.772	7.833	6.498	0.472
		Older	14	30	59.00	56.20	0.279	7.384	6.640	0.652
		Total	31	57	64.45	61.91	0.236	7.631	6.573	0.387
	LOAD	Total	68	28	48.44	50.61	0.281	11.326	11.273	0.967
FHL2 c2	GD	Younger	44	39	*33.61*	*36.56*	*0.028*	*3.793*	*8.026*	*0.001*
		Older	47	33	56.98	57.70	0.586	8.031	8.023	0.994
		Total	91	72	45.68	46.25	0.776	*5.982*	*8.025*	*0.012*
	EOAD	Younger	17	27	41.59	41.30	0.900	8.836	8.012	0.709
		Older	14	30	48.57	51.93	0.119	9.047	5.539	0.215
		Total	31	57	44.74	46.89	0.256	8.931	6.711	0.198
	LOAD	Total	68	28	60.60	60.43	0.889	10.243	8.357	0.060
TRIM59 c7	GD	Younger	44	39	*30.43*	*26.24*	*0.0001*	*8.609*	*4.944*	*0.003*
		Older	47	33	49.60	49.94	0.866	9.663	9.734	0.965
		Total	91	72	40.33	37.25	0.132	9.153	7.170	0.056
	EOAD	Younger	17	27	*40.76*	*35.00*	*0.004*	*12.176*	*5.653*	*0.008*
		Older	14	30	*48.93*	*44.23*	*0.013*	6.805	7.264	0.803
		Total	31	57	*44.45*	*39.86*	*0.006*	*9.751*	*6.500*	*0.023*
	LOAD	Total	68	28	54.32	52.86	0.385	9.970	10.033	0.955
KLF14 c1	GD	Younger	44	39	4.20	4.56	0.261	*5.082*	*8.873*	*0.002*
		Older	47	33	10.11	11.18	0.083	12.101	10.751	0.414
		Total	91	72	7.25	7.60	0.564	8.707	9.734	0.344
	EOAD	Younger	17	27	*10.18*	*6.74*	*0.013*	14.478	9.076	0.096
		Older	14	30	8.07	9.43	0.206	*14.366*	*9.714*	*0.035*
		Total	31	57	9.23	8.16	0.239	*14.427*	*9.415*	*0.016*
	LOAD	Total	68	28	13.24	11.89	0.102	13.247	12.234	0.653

Significant values marked with italics

#### Validation of predictive capacity of single DNAm markers in the tested disease groups

In order to evaluate the predictive capacity of particular age-related DNAm markers in the tested disease groups, separate prediction models for five selected CpGs were developed with 305 healthy individuals described in our previous study used as a training set [11]. In the present study, artificial neural network (ANN) approach was applied for prediction modeling instead of linear regression used before. ANN is a mathematical representation of the human neural architecture. It is composed of multiple nodes, called neurons, connected by links, which have assigned weights expressing the strength of the connections. Weights are adjusted in the process of neural network learning [50]. ANN models were developed in the form of multilayer perceptron (MLP) with one hidden layer and an automatically selected number of neurons (between 1 and 50). The activation functions were hyperbolic tangent for the hidden layer and identity for the output layer. For the remaining settings, default initial parameters of IBM SPSS Statistics were applied. The developed ANN prediction models for single DNAm markers were tested using groups of EOAD, LOAD, and GD patients and age-matched healthy controls. Predicted age of individuals was compared with the true chronological age of individuals for the calculation of mean absolute error (MAE). Independent sample Student’s *t* test was used to compare mean predicted age and MAE calculated for the particular disease groups with age-matched healthy individuals. All the analyses were performed using PS IMAGO 4 (IBM SPSS Statistics 24).

### Multivariate ANN prediction model

Finally, multivariate prediction model was developed based on all five selected age-related DNAm markers. Similarly to single CpG prediction models, 305 healthy individuals were used as a training set [11] and neural network approach was used for prediction modeling. Developed prediction model was tested using the same disease groups and age-matched healthy controls. Performance of the developed prediction model was evaluated throughout the calculation of MAE of predicted and chronological age and additionally percentage of correct predictions. Predictions were considered correct when the difference between actual and predicted age did not exceed ±5 years. This cutoff value was set according to the standard error of estimate calculated for the model to be at the level of~4.5 years [11]. Analyses were performed using PS IMAGO 4 (IBM SPSS Statistics 24).

## Results

### DNA methylation status and predictive capacity of the single DNAm markers in the tested disease groups

Three tested disease groups comprising of 31 early onset Alzheimer’s disease patients, 68 late onset Alzheimer’s disease patients, and 91 Graves’ disease patients (Table 1) were used in this study to assess DNA methylation profile and predictive performance of five selected age-related CpG sites (ELOVL2 c7, C1orf132 c1, FHL2 c2, TRIM59 c7, KLF14 c1) in these disease groups. The results were compared to the age-matched healthy controls. All the selected markers were found to have unchanged DNA methylation status and age prediction capacity in the group of late onset Alzheimer’s disease patients (Table 2).

**Fig. 1:**
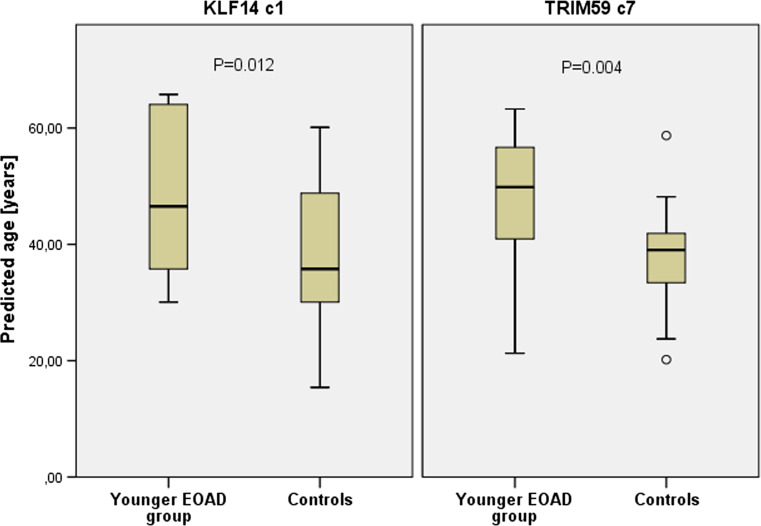
Predicted age in younger EOAD group and age-matched healthy controls. Prediction analysis was performed with KLF14 c1 and TRIM59 c7 markers

Early onset Alzheimer’s disease patients showed accelerated hypermethylation of TRIM59 c7 marker (*P* = 0.006), and the effect was most significant in younger EOAD group (*P* = 0.004). Aberrant hypermethylation of TRIM59 c7 led to decreased age prediction accuracy in EOAD patients, and again, the effect was most significant in younger EOAD group with MAE = 12.2 calculated for patients and MAE = 5.7 assigned for age-matched healthy controls (*P* = 0.008). When TRIM59 c7-based predicted age was compared, patients in younger EOAD group were found to have significantly increased age (9 years on average) when comparing to age-matched healthy controls (*P* = 0.004, Fig. 1). Increased methylation was also found in younger EOAD group for KLF14 c1 (*P* = 0.013, Table 2). Although no prediction accuracy deviation expressed by MAE was observed (*P* = 0.096) for patients included in younger EOAD group, they were found to have significantly increased age (by 10.1 years) when predicted age of patients and controls was directly compared (*P* = 0.012, Fig. 1). Marginal deviation in MAE was observed for older EOAD group (*P* = 0.035), but difference was insignificant when predicted age was compared (*P* = 0.093).

**Fig. 2:**
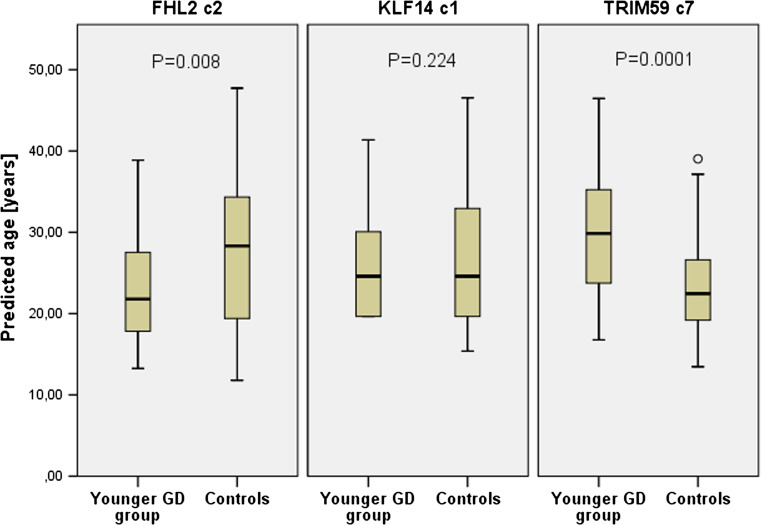
Predicted age in younger GD group and age-matched healthy controls. Prediction analysis was performed with FHL2, KLF14 c1, and TRIM59 c7 markers

Aberrant DNA methylation pattern was also observed in the group of GD patients. Similarly to EOAD patients, accelerated hypermethylation was observed for TRIM59 c7. The effect was significant in younger GD group (*P* = 0.0001, Table 2). Prediction analysis performed using TRIM59 c7 marker showed significantly decreased age prediction accuracy in younger GD group (MAE = 8.6) when comparing to healthy controls (MAE = 4.9, *P* = 0.003) with younger GD patients predicted to be 6 years older than controls (*P* = 0.0001, Fig. 2). In contrast, decreased methylation was noticed for younger GD group when FHL2 c2 marker was analyzed (*P* = 0.028). Significant difference in MAE was observed between younger GD group (MAE = 3.8) and controls (MAE = 8, *P* = 0.001), and patients in this group were predicted 4.9 years younger than controls (*P* = 0.008, Fig. 2). Ambiguous result was obtained for KLF14 c1 marker. Although significantly decreased age prediction accuracy measured by MAE was observed in younger GD group comparing to control group (*P* = 0.002), no significant difference was observed in DNA methylation status (*P* = 0.261) and no significant difference in mean predicted age was noted (*P* = 0.224, Fig. 2).

Unchanged DNA methylation status and predictive performance were detected for the two remaining markers, ELOVL2 c7 and C1orf132 c1 in the three disease groups and all age group categories.

### Prediction modeling using five DNAm loci

**Fig. 3:**
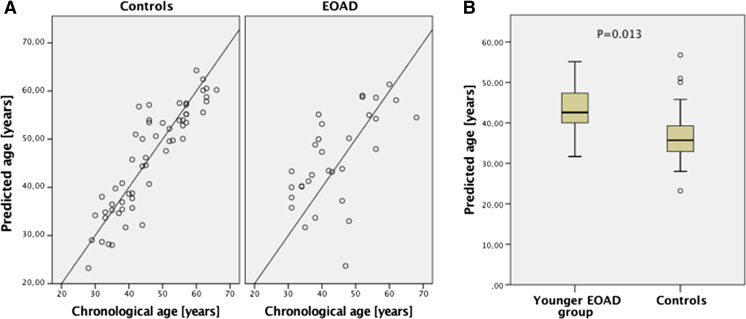
Age prediction analysis in EOAD patients using multivariate ANN model. **a** Predicted vs chronological age in total EOAD group and age-matched healthy controls. **b** Predicted age comparison between younger EOAD group and age-matched healthy controls

In the next step, multivariate prediction model was developed including all five age-related CpGs. Developed multivariate ANN model predicted age of total EOAD patients with significantly decreased accuracy (MAE = 7.1) when comparing to the healthy control group (MAE = 3.8, *P* = 0.002, Table 3). When different age categories were analyzed, this effect was observed only in younger EOAD group (*P* = 0.011). The number of correct predictions was also decreased in the total EOAD group (38.7%) when comparing to the healthy controls (70.2%), and again, the effect was noticeable particularly in younger EOAD group (Table 3). On average, patients in the total EOAD group were predicted to be 1.7 years older than the chronological age of patients but 6.4 years older than the chronological age of patients when only younger EOAD group was taken into account (Fig. 3a). In contrast, healthy controls were predicted to be on average 0.38 years younger than the true chronological age of individuals, and prediction accuracy did not dropped when only younger individuals (below 45 years old) were taken into account (predicted 0.12 years younger than the true chronological age, Fig. 3a). When predicted age was compared between patients and controls, patients included in younger EOAD group were predicted to be at significantly higher age (by 5.8 years) than matched controls (*P* = 0.013, Fig. 3b).

**Table 3:** MAE and percentage of correct predictions in EOAD, LOAD, and GD patients compared to age-matched healthy controls

Disease group	Age category	Parameter	Patients	Controls	*P* value
GD	Younger	MAE	2.5	2.7	0.595
		Correct predictions (%)	93.2 (41/44)	87.2 (34/39)	
	Older	MAE	6.1	4.4	0.106
		Correct predictions (%)	55.3 (26/47)	60.6 (20/33)	
	Total	MAE	4.4	3.5	0.146
		Correct predictions (%)	73.6 (67/91)	75.0 (54/72)	
EOAD	Younger	MAE	7.3	4.2	*0.011*
		Correct predictions (%)	29.4 (5/17)	66.7 (18/27)	
	Older	MAE	7.0	3.4	0.066
		Correct predictions (%)	50.0 (7/14)	73.3 (22/30)	
	Total	MAE	7.1	3.8	*0.002*
		Correct predictions (%)	38.7 (12/31)	70.2 (40/57)	
LOAD	Total	MAE	6.1	5.6	0.382
		Correct predictions (%)	76.5 (52/68)	50.0 (14/28)	

Prediction analysis performed with multivariate ANN model. Significant values marked with italics

No deviation in MAE and mean predicted age was noted in the remaining study groups, that is LOAD and GD patients when multivariate NN prediction model was applied (Table 3).

## Discussion

Difference between chronological and biological age of an individual is important in medical and forensic studies. Biological age is relevant for onset and progression rate of many diseases. It has been suggested that delaying the rate of biological aging may have a positive overall impact on the life expectancy and quality [20, 21]. In forensics, prediction of chronological and biological age is important. Besides direct use of chronological age for intelligence purposes, information about biological age can strengthen prediction of progressive appearance traits, which due to a recent progress in genetics can be predicted from DNA left at the crime scene. Differentiation between DNA methylation markers, which depend solely on the number of cell replications and those modified by other factors, may be important for developing more accurate models predicting chronological age in forensics. Evaluation of age markers may be accomplished by studying their response to various environmental factors and diseases that may change DNA methylation affecting accuracy of prediction of calendar age. Such markers should be avoided in prediction models aiming at prediction of chronological age.

### Validation of age predictive markers

In this research study, three groups of patients, EOAD, LOAD, and GD, were investigated using pyrosequencing technology and ANN prediction model based on five previously selected age-correlated CpG sites in ELOVL2, C1orf132, KLF14, FHL2, and TRIM59. Significant differences of age prediction capacity were noted for particular markers in the case of EOAD and GD patients, and they influenced the overall age prediction in EOAD group. The effects were more obvious in the younger groups of investigated patients, and these may be caused by the previously described general deregulation of age-related DNA methylation markers in elderly individuals [11, 13].

The overall accuracy of multivariate ANN age prediction model was significantly decreased in EOAD patients as measured by MAE and the number of positive predictions. The mean predicted age of patients was higher than the true chronological age of patients and higher than the mean age of age-matched healthy controls. The remaining study groups of LOAD and GD patients were predicted with accuracy observed in the age-matched healthy controls when multivariate ANN age prediction model was applied.

In order to get a better insight into age prediction accuracy of the model, separate examination of prediction accuracy of single five age predictors was undertaken in the three investigated groups of individuals. The analysis disclosed significant loss of accuracy as measured by MAE in the case of TRIM59, KLF14, and FHL2. The changed prediction capacity was noted not only in the group of EOAD but also in the younger individuals suffering from GD. TRIM59 and KLF14 were found to be hypermethylated in EOAD patients indicating older age of the investigated individuals. Thus, the worse performance of the overall prediction model was caused by deregulation of DNA methylation in these two loci. Interestingly, TRIM59 was also hypermethylated in patients included in the younger GD group, but this effect was not observed when investigating the overall performance of the model, because of the balancing influence of hypomethylation in the FHL2 locus.

Alzheimer’s disease is the most common type of dementia characterized by massive neuronal loss caused by overproduction of β-amyloid and hyperphosphorylated microtubule-associated protein tau accumulated into senile plaques and neurofibrillary tangles, respectively. Senile plaques with minimal cortical tau pathology and no accompanying history of cognitive decline are also hallmarks of pathological aging. Moreover, β-amyloid accumulation in both conditions is remarkably similar [22]. There are two main types of AD: EOAD and LOAD. Familial EOAD represents 1–5% of all cases of AD and in 40% is associated with mutations in the genes *PSEN1*, *PSEN2*, and *APP*. The group of EOAD patients studied here has been well characterized genetically [23–25]. The genetics of LOAD is still poorly understood, but significant progress has been made by the GWAS analyses, which have identified 25 genes to be associated with this type of AD [26]. The involvement of DNA methylation in AD is still under debate, especially its causal or subsequent role remains unclear [27]. However, growing evidence shows AD as being associated with DNA hypermethylation and histone deacetylation, suggesting a general repressed chromatin state and epigenetically reduced plasticity in AD [28]. Generally, analysis of DNA methylation in AD patients revealed differences in brain tissue, but results for peripheral blood are conflicting [29, 30]. In a medical sense, our result on methylation differences in aging markers further emphasizes differences between the two groups of Alzheimer’s disease patients.

GD is an autoimmune disorder, which affects up to 2% of the European population. Antibody-driven activation of the thyrotropin receptor leads to hyperfunction of the thyroid gland and thyroid enlargement and in consequence increased production of the thyroid hormone [31]. Previous study investigated T cell receptor rearrangement excision circle (sjTREC) concentration in Graves’ disease patients and reported that although generally decreasing with age, it was significantly higher in GD group compared with controls [32]. Examination of the decrease in the number of sjTREC molecules caused by thymus involution occurring in the course of human life has a predictive capacity and has been implemented in forensic age prediction [33]. Our investigation of age predictive DNA methylation markers does not support a conclusion that can be drawn from the study performed by Strawa about younger age of GD patients [32]. A hypomethylation of the FHL2 gene predicts younger age of patients, but two other markers (TRIM59 and KLF14) have increased methylation comparing to the controls, while ELOVL2 and C1orf132 are unchanged.

The FHL2 encodes a multifunctional adaptor protein that is involved in the regulation of gene transcription, signal transduction, and cell proliferation and differentiation [34]. Interestingly, FHL2 may act depending on a tissue as an oncoprotein or as a tumor suppressor [35]. It has been reported that FHL2 is involved in colorectal, gastric, and pancreatic cancer and hepatocellular carcinoma [36, 37]. The FHL2 protein was found to interact with presenilin 1 and presenilin 2, both involved in AD [38, 39].

The TRIM59 gene was hypermethylated in both EOAD and GD patients. This gene is involved in cancer that has been suggested as multitumor marker detecting early tumorigenesis [40]. TRIM59, which encodes an ubiquitin ligase, might be involved in neurodegeneration process by affected proteostasis, for instance by contribution to accumulation of neurofilament light chain, similarly to TRIM2 [41]. Several data suggested pro-apoptotic cooperation of p53 and TRIM59, and activation of p53 signaling is commonly known to leading to death of post-mitotic neurons in Alzheimer’s disease patients. A physical interaction of TRIM59 and p53 under TRIM59 upregulation resulted with ubiquitination and degradation of p53 [42].

The methylation marker KLF14 was found to have decreased age prediction accuracy in EOAD and GD patients. However, the results were ambiguous as no significant change in DNA methylation was observed in particular age group categories, and therefore, this effect needs to be further validated on a larger sample size. Loss of KLF14 in mouse was shown to cause centrosome amplification and tumorigenesis, and it has been suggested that reduction of KLF14 may increase the risk of breast cancer and colon cancer [43]. KLF family was found to be involved in transcriptional modulation of neuronal genes, e.g., dopamine D2 receptor [44]. Importantly, KLF14 reduction was reported to be responsible for aneuploidy and finally tumorigenesis [43]. It is very likely that this could be linked with increased DNA damage stress response, commonly observed in AD, that could be further linked with p53-mediated post-mitotic apoptosis, as also proposed by [45].

It is worth noting that age-associated changes in DNA methylation patterns may have regulatory role on gene activity and developmental processes. Recently, it has been shown that the mean age-associated DNA methylation patterns manifests in early childhood (2–16 years) and the majority of age-associated loci involved increased DNA methylation resulting in decreased gene expression [46]. Authors concluded that the results could pinpoint genes susceptible to aging-related disease-associated epigenetic dysregulation.

Markers epigenetically regulated in diseases should be avoided in prediction models developed for estimation of chronological age. However, it seems that each DNA methylation age predictor may have a unique sensitivity to various external factors. Our recent study involving the same prediction model discovered significantly lower predicted age of patients after hematopoietic stem cell transplantation (HSCT) [47]. This result was discordant to the report of Weidner et al., and detailed analysis of our results showed that the observed discrepancy is caused by different sets of predictors used in both studies [48]. In particular, the effect observed in our study was a result of hypermethylation of a single C1orf132 locus, while the remaining loci included in the model did not show this pattern. It means that in some cases, like HSCT, C1orf132, which encodes for long noncoding RNA MIR29B2C, may not be a good predictor of chronological age.

However, in the present research study, C1orf132 and ELOVL2 were found to be stable age markers in three groups of patients. It is worth noting that HSCT is a very rare medical treatment (~50,000 worldwide HSCTs in 2006), while the incidence of GD is 20 to 50 cases per year, per 100,000 individuals [49, 50]. ELOVL2 is the most thoroughly validated age marker [4, 8, 11, 51]. Horvath demonstrated that cell passaging in general increases DNA methylation age, but he also rejected the hypothesis that DNA methylation age is the same as mitotic age (the number of cell divisions) as it tracks chronological age in cell types which not proliferate. Therefore, Horvath proposed that DNA methylation reflects rather the work done by an organism to maintain epigenetic stability [52]. On the other hand, Bacalini et al. have reported differences in age-associated methylation change schemes in two known age predictors: ELOVL2 and FHL2. The study showed that ELOVL2 methylation is a marker of cell divisions occurring during human aging and not the senescence [17]. Contrary, DNA methylation of the FHL2 gene was not strictly associated to cell divisions in that research. This means that DNA methylation in different loci may indeed depend on various factors, and there is a chance that some predictors may better measure chronological age (mitotic age) than the others. The study of Bacallini shows that methylation of FHL2 can be affected by other age-associated factors, that is also supported by our study of GD patients. Our study may help in selection of the most optimal marker set for chronological age prediction in forensics, but due to a small number of samples in particular disease age groups, the findings need to be replicated. Overall, the study reinforces the significance of ELOVL2 and C1orf132 as predictors of chronological age in forensic investigations. Further studies exploring other potential confounders of DNA methylation will be particularly interesting in the case of C1orf132 that is a very powerful age predictor. This locus encodes for a long noncoding RNA and may play a role in epigenetic regulation. As mentioned earlier, our previous study demonstrated its potential involvement in graft function after HSCT [47].

### Prediction modeling using artificial neural network approach

The artificial neural networks used in the study for prediction modeling seem to be a good alternative for the traditional parametric methods like linear regression. ANN can be a simple solution to eliminate a problem of nonlinear patterns that can be attributed to particular DNAm markers [53]. Many authors highlight different advantages of neural networks: the ability to recognize and learn all types of relations, lack of assumptions on the distribution of the input data, automatic handling of variable interactions, or the high tolerance to missing and noisy data [54–57]. We demonstrated its superiority over regression methods in our previous reports on hair morphology [2] and eye color prediction [3]. Recently, ANN has also been shown to be more accurate in age prediction modeling [58, 59]. For comparison purposes, calculations in the present study were performed with linear regression confirming the general outcome of the study but demonstrating slightly lower prediction accuracy parameters when comparing to the neural networks approach (data not present).

### Forensic impact of the study

The multivariate ANN age prediction model failed to predict age accurately in case of EOAD patients. Since EOAD is a very rare condition that affects merely 1–5% of AD patients, this finding has a minor impact on forensic age prediction. However, separate analyses of prediction capacity of five markers included in the evaluated model clearly showed that three markers—TRIM59, KLF14, and FHL2—revealed altered DNA methylation patterns in EOAD and GD groups. Although this finding does not disqualify the three markers as predictors of chronological age, further studies shall investigate their sensitivity to various factors affecting biological age of an individual. Importantly, our study showed that the two important age predictors—ELOVL2 and C1orf132—keep their high predictive capacity in three groups of patients. Our finding confirms conclusion from a recent study reporting DNA methylation in ELOVL2 to depend solely on the number of cell divisions and FHL2 to depend on other age-related factors [17]. This study shows that C1orf132, a very powerful age predictor, may together with ELOVL2 comprise a good foundation for prediction of chronological age in forensics, although further studies are necessary especially for C1orf132 since in our previous investigation, this locus showed changed DNA methylation in the group of individuals after HSCT.
